# Supplementary material for: Self‐Adaptive Quantum Kernel Principal Component Analysis for Compact Readout of Chemiresistive Sensor Arrays
Source: Adv Sci (Weinh). 2025 Jan 23;12(15):2411573. doi: 10.1002/advs.202411573 (PMC12005759; doi:10.1002/advs.202411573)
Supplement: Supplementary file 1 — Supporting Information [file ADVS-12-2411573-s001.docx]

**Supporting Information**

**Self-Adaptive Quantum Kernel Principal Components Analysis for Compact Readout of Chemiresistive Sensor Arrays**

Zeheng Wang **^1, 2, †^**, Timothy van der Laan **^2^**, and Muhammad Usman **^1,3^**

**^1^** Data61, CSIRO, Clayton, VIC 3168, Australia

**^2^** Manufacturing, CSIRO, West Lindfield, NSW 2070, Australia

^3^School of Physics, The University of Melbourne, Parkville, VIC 3010, Australia

**† Corresponding author. Email: zenwang@outlook.com**

Keywords: Quantum computing; Quantum PCA; Quantum Machine Learning; Chemiresistive sensor; IoT

| **Supplementary Algorithm S1**: Quantum kernel training with self-adaptive parameters  The SAQK is realized by a pre-training process, where the training set is used to train the variational quantum circuit layer consisting of a $R_{z}$ gates layer for feature mapping and a parameterized $R_{x}$ gates layer for the kernel’s adaptivity. | |
| --- | --- |
| Input:  - Classical dataset {$x_{i},y_{i}$} for i = 1 to $N$  - Initial parameters $\theta_{0}$  - Feature map $U_{F} \left( \boldsymbol{x} \right)$ ($R_{z}$ rotation-based encoding)  - Trainable circuit $U_{V}^{\theta} \left( \boldsymbol{x},\boldsymbol{\lambda} \right)$ ($R_{x}$ rotation-based adaptability)  - Optimizer (in this study, SPSA)  - Maximum iterations $T$  Output:  - Optimized parameters $\theta^{*}$ | |
|  | **Procedure** |
|  | **Initialize** $\theta$ ← $\theta_{0}$  **for** t = 1 to $T$**do**  // Step 1: Compute the quantum kernel matrix  **for** i = 1 to $N$ **do**  **for** j = 1 to $N$ **do**  Compute $U_{F} \left( \boldsymbol{x} \right)\cdot{H\cdot U}_{V}^{\theta} \left( \boldsymbol{x},\boldsymbol{\theta} \right)$  Compute $K\left( x_{i},x_{j} \right)$  // Step 2: Evaluate the loss function  Compute $Loss\left( \theta\right)$: Defined by SVC Loss  // Step 3: Update the parameters by step $\eta$:  $\theta\leftarrow\theta-\eta\nabla_{\theta}Loss\left( \theta\right)$  // Step 4: Check for convergence  **If** $Loss\left( \theta\right)$ < threshold **then**  **break**  **end** **for**  **Return** $\theta^{*}$  Construct rotation gates $U_{F} \left( \boldsymbol{x} \right)\cdot U_{V}^{\theta} \left( \boldsymbol{x},\boldsymbol{\theta} \right)$  **End**. |

| **Supplementary Algorithm S2**: Preprocessing pipeline for data encoding and normalization | |
| --- | --- |
| Input:  - Raw dataset: A CSV file containing sensors’ current values and a label column.  - Selected sensors: A list of the top N important sensors by method in Ref^[1]^.  - Normalization range: [-1, 1] (using MinMaxScaler method of Scikit-Learn package).  Output:  - Encoded labels: Integer-encoded labels stored in a new column.  - Normalized sensors’ values: Current values scaled to the range [-1, 1]. | |
|  | **Procedure** |
|  | Load the raw dataset into a DataFrame.    Encode the labels:  a. Extract the label column.  b. Initialize a label encoder and transform the labels into integers.  c. Add the encoded labels to the original dataset as a new column.  d. Reorder columns to place encoded labels as the first column.  e. Save the updated dataset with encoded labels to a new CSV file.  Normalize the features:  a. Load the top N important sensors.  b. Select the corresponding current value columns from the dataset.  c. Initialize a MinMaxScaler with the range [-1, 1].  d. Fit the scaler to the selected features and transform the values.  e. Convert the normalized data into a DataFrame.  Output the normalized features and labels for further processing.  **End**. |

| **Supplementary Algorithm S3**: t-SNE for 2D data visualization (implemented from Scikit-Learn package with default settings). | |
| --- | --- |
| Input:  - High-dimensional dataset $X\in\mathbb{R}^{n\times d}$, where $n$ is the number of samples and $d$ is the dimensionality.  Output:  - 2D embedding $Y\in\mathbb{R}^{n\times2}$ for visualization. | |
|  | **Procedure** |
|  | 1. Initialization: 2. Set target dimensionality $k=2$ for 2D embedding. 3. Define hyperparameters (default values used in scikit-learn)  - Perplexity $p=30$: Determines the balance between local and global structure. - Learning rate $lr=200$: Controls optimization speed. - Number of iterations $t=1000$: Determines the optimization steps.  1. Compute Affinities in High-Dimensional Space:   For each point $x_{i}\in X$, compute pairwise similarities using a Gaussian kernel. Then, Normalize the result $P_{ij}$to ensure conditional probabilities sum to 1 for each point.   1. Initialize Low-Dimensional Embedding:   Initialize $Y\in\mathbb{R}^{n\times2}$ randomly.   1. Optimize Embedding via Gradient Descent:   Minimize the Kullback-Leibler (KL) divergence between the high-dimensional probabilities $P_{ij}$ and low-dimensional similarities $Q_{ij}$. Then Compute $Q_{ij}$ using a Student’s t-distribution. Iteratively update $Y$ using gradient descent to minimize *C*$.$   1. Stop Criteria:   Continue until convergence (change in *C* is minimal) or after *t* iterations.   1. Output:   Return $Y$, the 2D embedding for visualization.  **End**. |

**
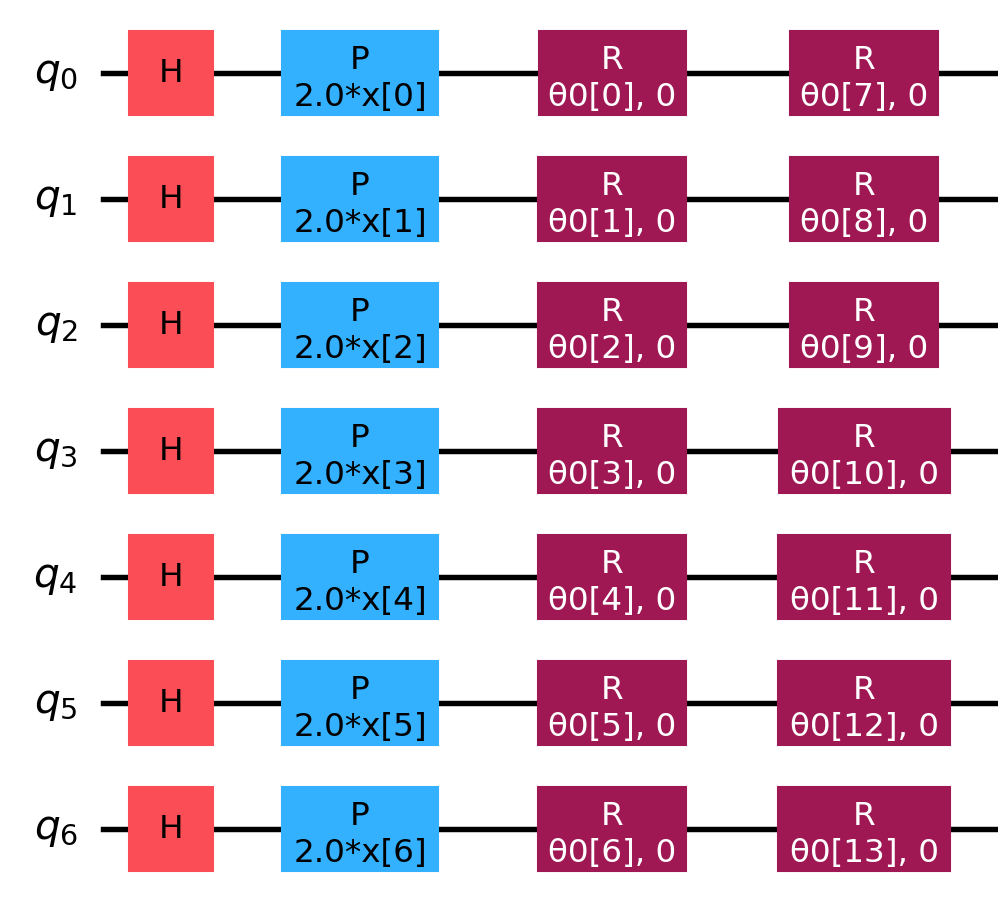
**

**Supplementary Figure S1.** The variational quantum circuit for the SAQK. The qubits are initialized to $\left. |0 \right\rangle^{7}$. The classical data $x_{i}$ controls the phase shift of the qubit by $exp(ix_{i}\boldsymbol{Z})$. The parameterized $R_{x}(\theta)$ gates are trainable. The Hadamard gates are introduced to increase the quantum coherence^[2,3]^.


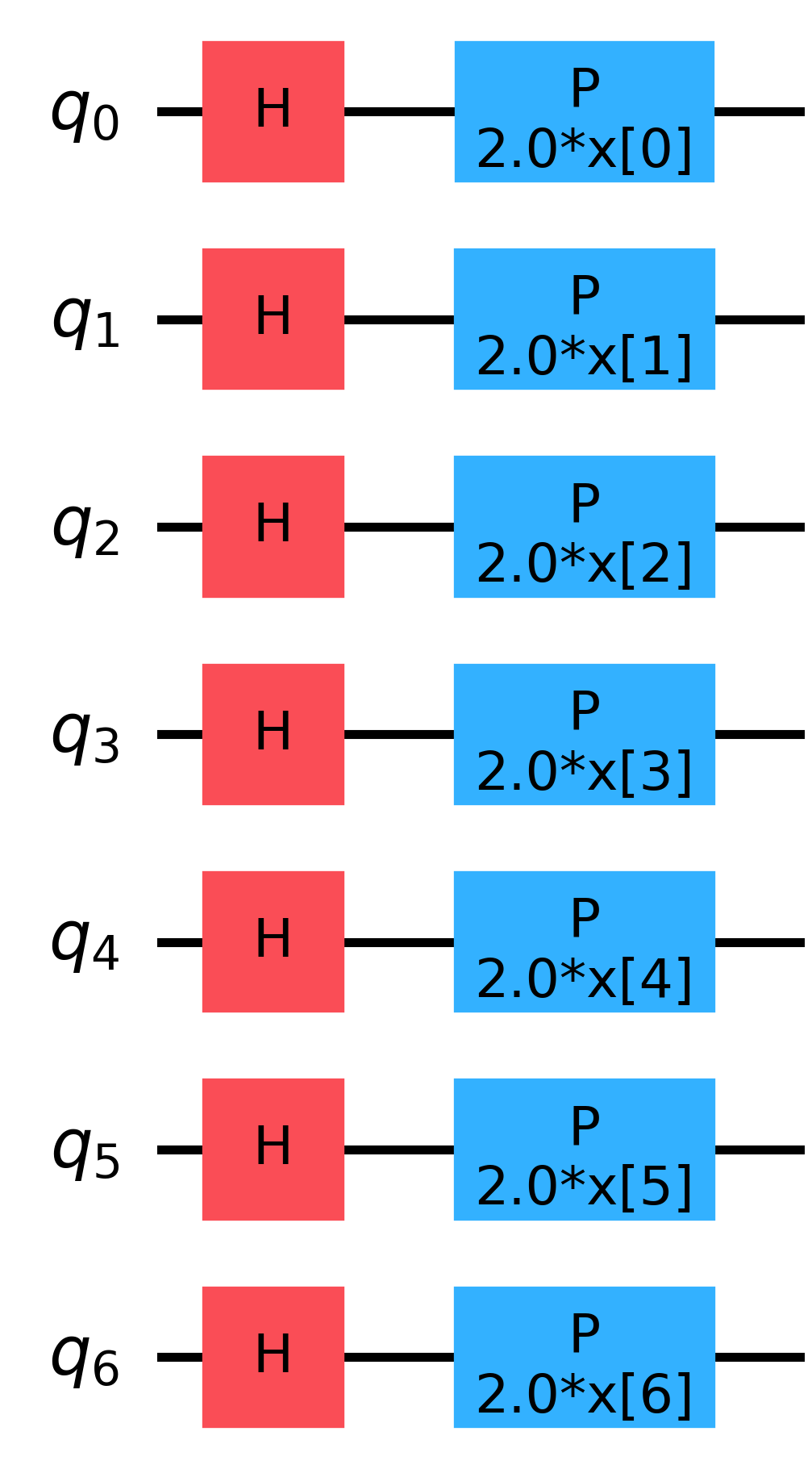


**Supplementary Figure S2.** The quantum circuit of Pauli-Z mapping. In this kernel method, the classical data $x_{i}$ controls the phase shift of the qubit. The qubits are initialized to $\left. |0 \right\rangle^{7}$.


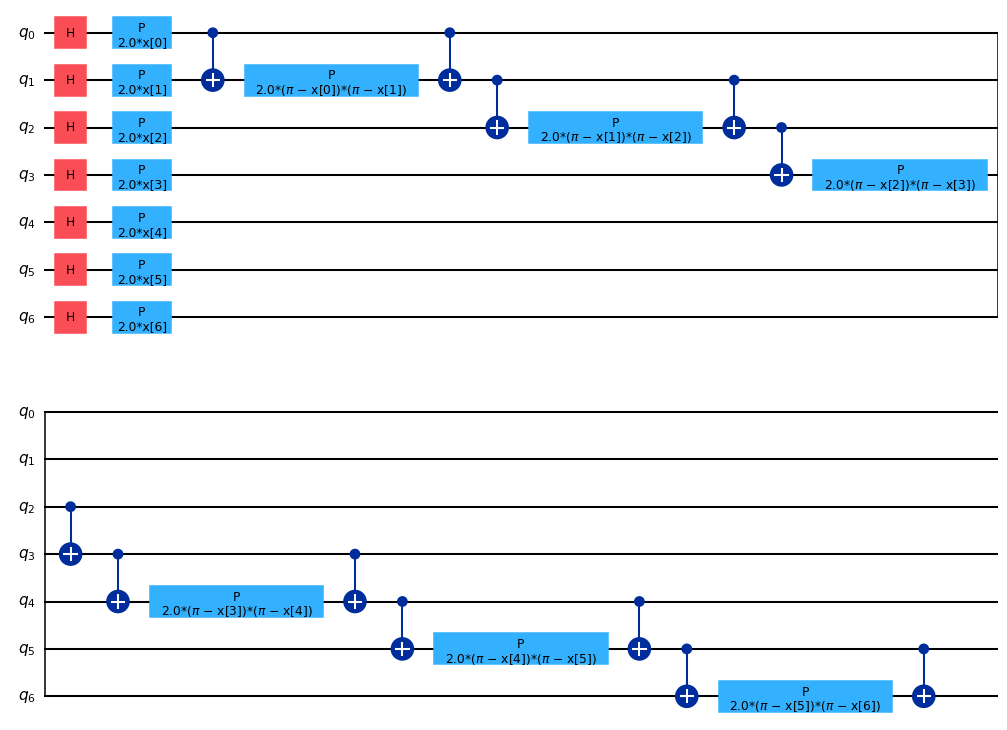


**Supplementary Figure S3.** The quantum circuit of ZZ-Feature mapping. The qubits are initialized to $\left. |0 \right\rangle^{7}$. Compared to the Pauli-Z mapping, this mapping incorporates entanglement between qubits through additional controlled-Z gates. The ZZ-Feature mapping enhances the representational power by introducing pairwise interactions between qubits, allowing the feature map to capture higher-order correlations between input features. The statistic feature mapping employed in the ZZ-Feature mapping improves upon the Pauli-Z mapping by capturing higher-order correlations through entanglement. Nevertheless, it lacks the adaptability of the SAQK framework. Unlike the static nature of the ZZ-Feature mapping, SAQK incorporates variational circuits with trainable parameters, such as $R_{x}(\theta)$, allowing the feature map to dynamically adjust to the underlying data distribution. This variational approach enhances the expressivity of the feature map and enables superior information retention, particularly for datasets with complex structures or group symmetries, where static mappings like the ZZ-Feature mapping are less effective.


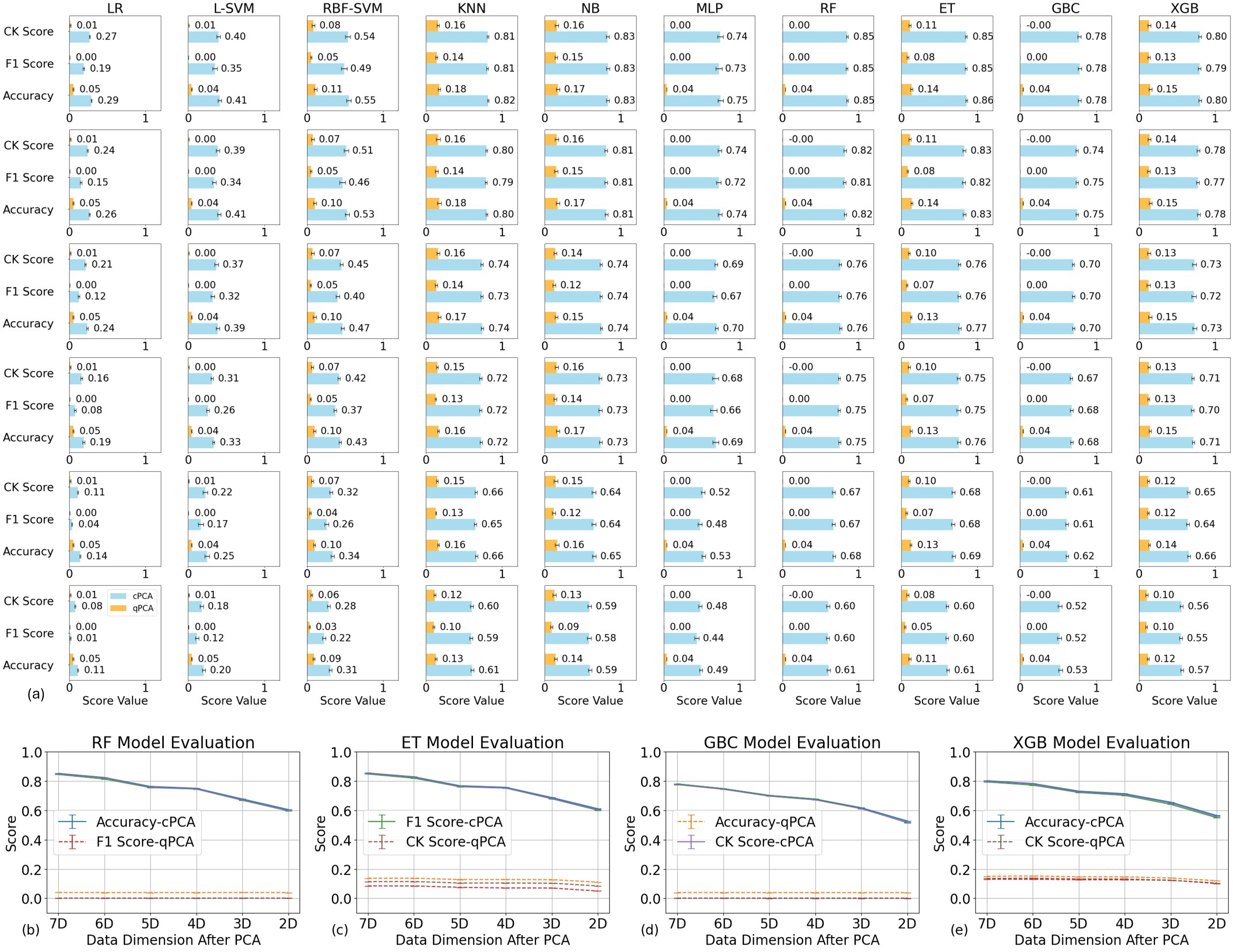


**Supplementary Figure S4.** Evaluation of the performance of dimensionality reduction using Pauli-Z kernel-based qPCA compared to the cPCA across various machine learning models. (a) Detailed comparisons of classification metrics, for ten machine learning algorithms. Across all models and metrics, the cPCA consistently outperforms the qPCA. (b)-(e) The evaluations on the ensemble-based classifiers (RF, ET, GBC, XGB), showing again that Pauli-Z kernel qPCA results in significantly lower Accuracy, F1 Score, and CK Score compared to cPCA as data dimensions are reduced. The trends suggest that the static Pauli-Z kernel struggles to adapt to diverse data structures, leading to poor classification performance. The findings underscore Pauli-Z kernel qPCA's limited ability to preserve meaningful features in the reduced dimensions, particularly for tasks requiring robust classification.


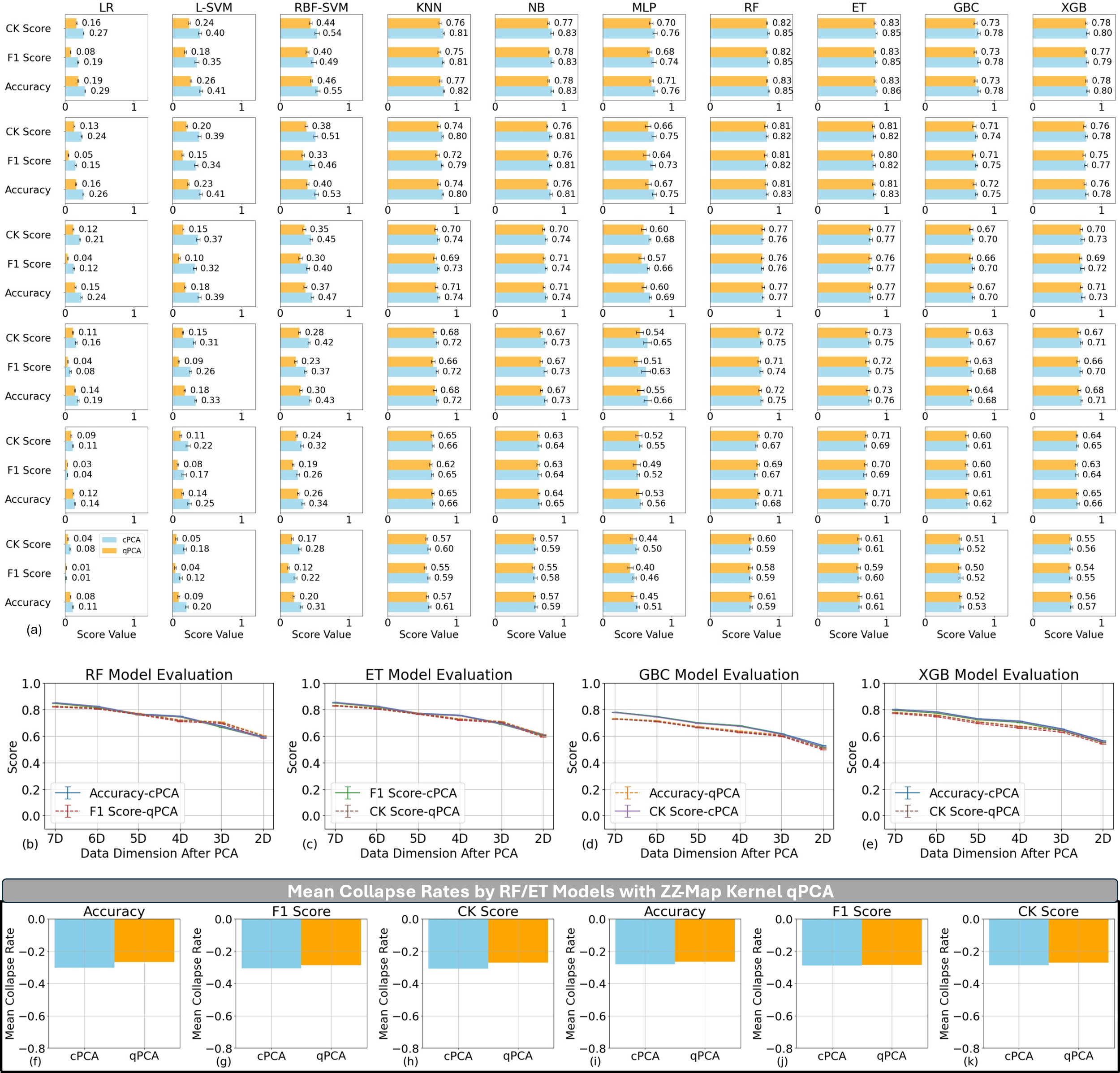


**Supplementary Figure S5.** Evaluation of dimensionality reduction performance using the ZZ-Feature Map kernel-based qPCA compared to cPCA across various machine learning models. (a) Detailed comparisons of classification metrics for ten machine learning algorithms. Compared to the Pauli-Z mapping kernel, the ZZ-Feature Map kernel demonstrates higher evaluation scores across several machine learning models, highlighting its improved feature extraction capability. (b)-(e) Performance trends of EL models, including RF, ET, GBC, and XGB, where the ZZ-Feature Map kernel achieves evaluation scores comparable to cPCA. (f)-(k) The mean collapse rates of evaluation scores for the two PCA methods across reduced dimensions. While the ZZ-Feature Map kernel achieves slightly lower performance than cPCA, it remains competitive.


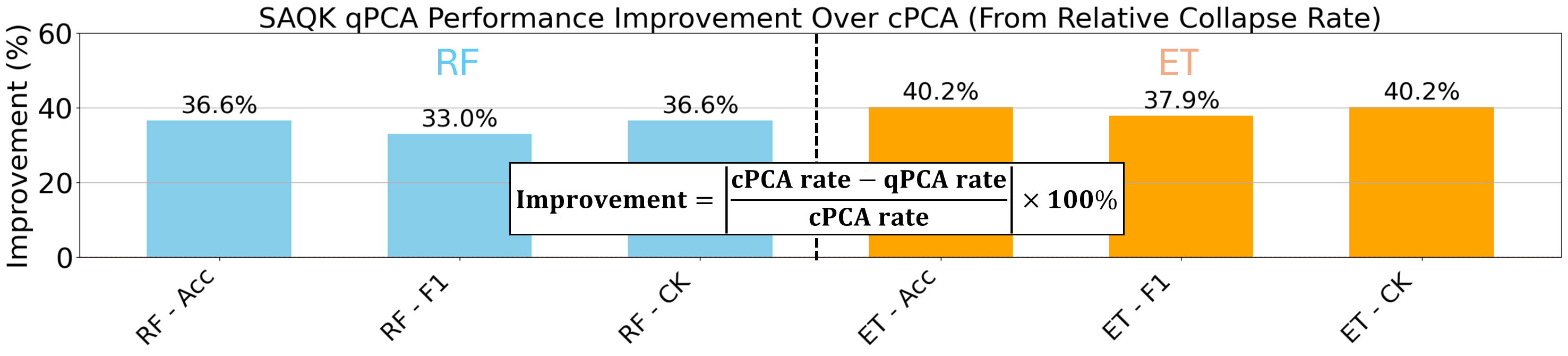


**Supplementary Figure S6.** Improvement of dimensionality reduction evaluation scores using the SAQK PCA compared to cPCA across high-performance EL models. The SAQK PCA consistently delivers over 30% improvement in relative collapse rates during dimensional reduction, with improvements reaching 36.6% for RF and 40.2% for ET in specific metrics. These results highlight the adaptive nature of SAQK PCA, which leverages its variational feature mapping to retain more critical information during dimensionality reduction, especially in scenarios where traditional cPCA struggles to preserve data structure. The significant gains further emphasize SAQK PCA's potential for enhancing classification performance in machine learning tasks involving complex datasets.

| Supplementary Table S1: Overview of dimensionality reduction techniques and their applications | | | | | |
| --- | --- | --- | --- | --- | --- |
| Method | Type | Scenarios | Strengths | Limitations | Ref. |
| PCA | Linear | Linear relationships data | Efficient, widely used, interpretable | Assumes linearity, sensitive to scaling of features | ^[4]^ |
| t-SNE | Nonlinear | Visualization of high-dimensional data into 2D or 3D | Excellent for visualization, captures local structures | Not suitable for supervised tasks | ^[5]^ |
| UMAP | Nonlinear | Visualization of high-dimensional data into 2D or 3D | Fast, preserves both local and global data structures | Less interpretable, parameter-sensitive | ^[6]^ |
| ICA | Linear | Feature separation in signal processing | Good for separating independent sources (e.g., in EEG, audio) | Assumes statistical independence of datasets | ^[7]^ |
| LDA | Nonlinear | Classification tasks where dimensionality reduction enhances class separability | Incorporates class information, improves separability | Assumes normally distributed data within classes | ^[8]^ |
| Autoencoders | Nonlinear | Large datasets with complex nonlinear structures | Learns hierarchical features, flexible for high-dimensional data | Requires extensive tuning | ^[9]^ |
| SAQK PCA | Nonlinear | Quantum feature space analysis for datasets with potential group-structured correlations | Captures group-structured correlations, more efficient for specific data | Mapping classical data into quantum space and relies on quality of quantum system | This work |

**Reference list of Supporting Information**

[1] Z. Wang, J. S. Cooper, M. Usman, T. Van Der Laan, *ACS Appl. Nano Mater.* **2024**, *7*, 24437.

[2] A. Streltsov, G. Adesso, M. B. Plenio, *Rev. Mod. Phys.* **2017**, *89*, 041003.

[3] H. Thomas, P.-E. Emeriau, E. Kashefi, H. Ollivier, U. Chabaud, *On the role of coherence for quantum computational advantage*, arXiv **2024**.

[4] Principal Component Analysis, Springer-Verlag, New York **2002**.

[5] T. T. Cai, R. Ma, *Theoretical Foundations of t-SNE for Visualizing High-Dimensional Clustered Data*, arXiv **2021**.

[6] L. McInnes, J. Healy, J. Melville, *UMAP: Uniform Manifold Approximation and Projection for Dimension Reduction*, arXiv **2018**.

[7] P. Comon, *Signal Processing* **1994**, *36*, 287.

[8] C. R. Rao, *Journal of the Royal Statistical Society Series B: Statistical Methodology* **1948**, *10*, 159.

[9] G. E. Hinton, R. R. Salakhutdinov, *Science* **2006**, *313*, 504.
